# Salve Journal of Functional Biomaterials, ad maiòra!

**DOI:** 10.3390/jfb1010001

**Published:** 2010-06-23

**Authors:** Francesco Puoci

**Affiliations:** *Founding Editor-in-Chief*, Dipartimento di Scienze Farmaceutiche, Università della Calabria 87036 Rende (CS), Italy; E-Mail: francesco.puoci@unical.it

The biomaterials field is one of the largest and fastest growing research areas both in the scientific community and in the industrial one. Biomaterials are the result of collaborations between different disciplines: chemistry, medicine, pharmacology, engineering and biology. The objective of this collaboration is to lead to the implementation of new devices to restore form and human body functions. The research on biomaterials reflects the constant need to replace or supplement human tissues and organs that have been physiologically compromised due to disease or traumatic events.

In this multi-faceted field of biomaterials, only in recent decades have significant and important results been achieved; thanks not only to the improvement of knowledge of physical, chemical and biological materials, but also to the implementation of innovative processes to synthesize biomaterials. The evolution of biomaterials is one of the bases of economic growth in the biomedical area and depends upon scientific progress. Thus, a strong link between scientific research and innovation is expected at the academic level, but also within firms. 

In 2008, biomaterial products reached a market size of 25.5 billion US dollars (USD), and the biomaterial device market size attained 115.4 billion USD. The biomaterial device market is expected to reach 252.7 billion USD by the year 2014. This massive revenue potential highlights the immense opportunities in the market. In the next five years, the biomaterials market is expected to grow at a compound annual growth rate of 15%. Around the world, companies in this field are rapidly increasing in size, and the key strategy for all of them is the same: innovation. 

On these premises, I am honored to introduce you – the reader and researcher – to this new journal: *Journal of Functional Biomaterials (JFB)*. The aim of *JFB* is to provide a forum for original research in all disciplines encompassed in our multidisciplinary field.

The mission of *JFB* is to focus the attention on physico-chemical characteristics and their importance in the interactions between biomaterials and living tissues. *JFB* is designed to consider for publication studies on the preparation, performance and use of biomaterials in biomedical devices, as well as their behavior in physiological environments.

The outcome of our focus on biomaterials will allow *JFB* to provide an unmatched integration of the various important aspects of biomaterial science. My commitment as Editor-in-Chief is to do my best to ensure that *Journal of Functional Biomaterials* is an excellent and advanced forum for both the scientific and technological communities. Please consider this a personal invitation to submit your contributions; I am very much looking forward to receiving your manuscript.

